# Alveolar Ridge Preservation After Tooth Extraction with DFDBA and Platelet Concentrates: A Radiographic Retrospective Study

**DOI:** 10.2174/1874210601711010099

**Published:** 2017-02-14

**Authors:** Behrang Baniasadi, Laurence Evrard

**Affiliations:** Department of Maxillofacial Surgery and Dentistry, Erasme Hospital, Université libre de Bruxelles, Brussels, Belgium

**Keywords:** Allografts, Alveolar bone loss, Extraction socket, Platelet rich fibrin, Ridge preservation, Socket preservation, Tissue regeneration

## Abstract

**Objectives:**

The purpose of this study was to evaluate vertical alveolar bone loss 3 months after tooth extraction when a technique of ridge preservation was applied using a particulate demineralized freeze-dried bone allograft 300 - 500 µm associated with platelet concentrates (platelet-rich-fibrin) in the form of gel and membranes.

**Material and Methods:**

A retrospective radiological clinical study was conducted on 56 patients for whom 95 extractions had been performed immediately followed by alveolar filling. Among the patients, 17 were smokers and 16 were provided with an immediate removable temporary prosthesis after extractions. Vertical bone loss was measured radiologically by panoramic X-ray before extractions and by a computed tomography scan 3 months after, at the level of mid-buccal bone wall, by two independent observers. For statistical analysis, Student’s t-test was performed to compare the mean bone loss between mono- and pluri-radicular teeth and to compare the mean bone loss between tobacco users *versus* non users and finally to compare the mean bone loss between individuals that had provisional removable prosthesis and those that had not.

**Results:**

Three months after tooth extraction, the mean of vertical loss of the mid-buccal bone wall was 0.72 (SD 0.71) mm (5.53% SD 5.19). No significant difference between bone loss at mono-radicular and pluri-radicular teeth (P = 0.982) was observed. There was no significant correlation between tobacco habits and bone loss (P = 0.2), nor between provisional removable prosthesis and bone loss (P = 0.786).

**Conclusion:**

These results indicate a good potential for the technique using Demineralized Freeze-Dried Bone Allograft 300 - 500 µm and platelet concentrates in alveolar bone preservation.

## INTRODUCTION

Post-extraction bone resorption is a progressive and irreversible process that has been well described in the scientific literature. A physiological alveolar bone resorption may reach up to 40% in height and 60% in width, with the gross loss being reached within 3 months after extraction [1]. Insufficient bone may compromise dental implant treatment with a risk of injuring the anatomical structures. Therefore, adequate alveolar ridge preservation is essential for an esthetical outcome and correct implant placement [2, 3].

Among the biomaterials used for post-extraction alveolar filling, allogenic bone has been described as a suitable material [4-7]. In particulate form, freeze dried bone allograft (FDBA) and demineralized freeze-dried bone allograft (DFDBA) are used in dental surgery and alveolar ridge preservation techniques [8-11]. It has been shown that when used in post-extraction sockets, allografts have a positive effect on height preservation [8]. In a histological study on alveolar preservation, it was shown that DFBDA leads to a statistically significant greater mean percentage of newly formed vital bone than FDBA [10].

The platelet concentrates (platelet-rich-fibrin) are obtained by blood centrifugation, following a method first described by Choukroun *et al.* [12]. The platelet concentrates contain a high concentration of growth factors (PDGF, TGF-β, IGF and VEGF) and anti-inflammatory molecules (IL-1β, IL-4, IL-6 and TNF-α) and enhance the healing process [13, 14]. This may lead to better bone repair and regeneration [14, 15]. It has been shown that platelet concentrates accelerate the healing of dermal soft tissue and of the oral mucosa in cases of dental extraction [16-18]. It is still unclear if platelet concentrates are able to accelerate bone healing and influence bone quality, although it has been suggested to occur in some studies related to extraction socket healing [19, 20].

In oral surgery, the clinical benefits of platelet concentrates, when combined with DFDBA in bone regeneration, have been suggested for the treatment of intrabony periodontal defects [21].

The aim of our study was to evaluate the potential benefits of a combination of DFDBA and platelet concentrates in alveolar preservation, measuring the amount of bone loss 3 months after extractions, in a great number of patients to compare with results obtained from other techniques.

## MATERIAL AND METHODS

The protocol of our study was accepted by the Ethical Committee of Erasme Hospital, (Université libre de Bruxelles, Brussels, Belgium) (P 2013/069).

A retrospective radiological clinical study was designed. The inclusion criteria were patients older than 18 years who had undergone dental extractions prior to implant treatment in our department. Their teeth had been extracted due to severe dental decay, fractures or periodontal disease.

No exclusion criteria were defined. Fifty-six patients were included in the study. The average age was 51-years-old, from 22 years and 84 years. Seventeen patients (30%) were smokers (> 10 cigarettes a day), and 16 patients (28%) were provided with a provisional removable prosthesis after extraction.

Ninety-five extractions were performed: 72 extractions of uni-radicular teeth (76%) and 23 extractions of pluri-radicular teeth (24%).

**Fig. (1):**
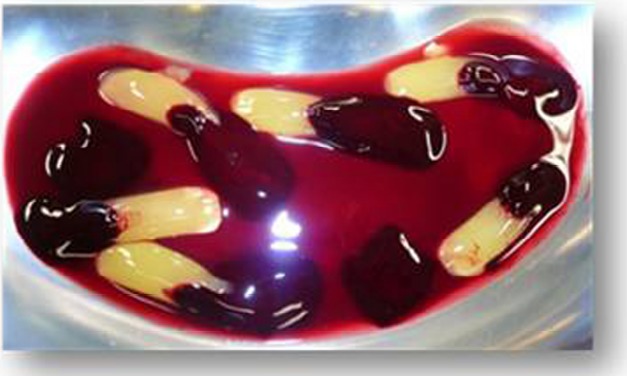
Platelets concentrates (platelet-rich-fibrin) were obtained by centrifugation of blood samples.

**Fig. (2):**
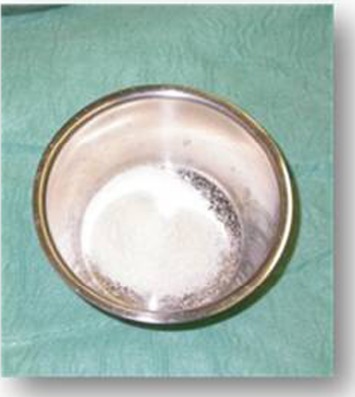
Demineralized freeze dried bone allograft 300 - 500 µm.

**Fig. (3):**
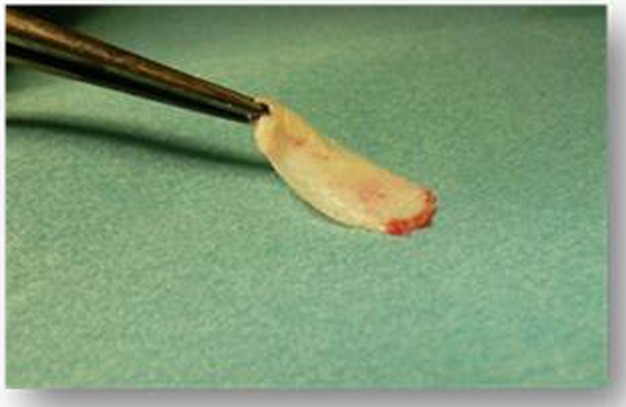
Parts rich in fibrin were pressed manually between gauzes in order to obtain autologous rich-in-fibrin membranes.

**Fig. (4):**
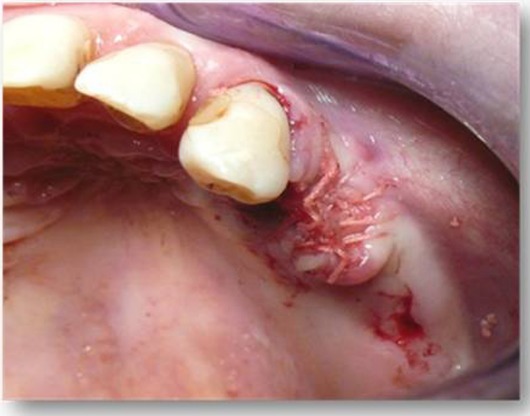
Closure of the sockets was realized using autologous fibrin membrane after filling the socket with a mixture of Demineralized freeze dried bone allograft. A polyglactin absorbable suture is used to close the socket.

At the beginning of surgery, platelet concentrates (platelet-rich-fibrin) were obtained by centrifuging patient blood samples in 10-ml tubes with no anticoagulant adjuvant at 3000 rpm for 10 minutes following a protocol described previously [22]. A portion of the centrifuged blood rich in platelets (named buffy-coats) was cut and mixed with particulate DFDBA (produced in Hôpital Erasme- ULB- Brussels) 300-500 µm (Figs. **1** and **2**). A portion rich in fibrin was pressed manually between gauze to obtain autologous rich-in-fibrin membranes (Fig. **3**). Atraumatic extractions were realized, and immediately after extraction, the socket was filled with a mixture of DFDBA and platelet concentrates. Closure of the sockets was realized using an autologous fibrin membrane to cover the filled socket and a polyglactin absorbable suture to close the socket (Fig. **4**).

For ethical reasons, no control group was used in this study, because no alveolar preservation leads to consequent bone loss [1-3].

In the post-operative period, ibuprofen 600 mg three times a day and paracetamol 500 mg were taken by the patients in case of pain. All patients received 1 gr of Amoxicillin twice a day for 4 days post-operatively, or in case of a penicillin allergy, they received clindamycin 300 mg three times a day for 4 days.

The pre- and post-operative alveolar ridge heights were measured by two independent observers.

**Fig. (5):**
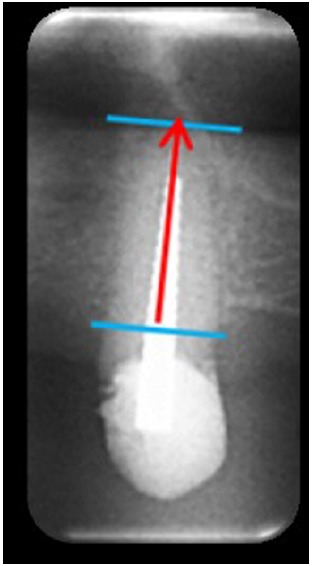
Pre-operative socket height is evaluated as the distance between apex and lowest crestal visible point on a panoramic radiography.

A panoramic radiograph of each patient was taken preoperatively. The panoramic radiographs were taken with Planmeca ProMax^®^ (precision = 0.1 mm) and analyzed with ROMEXIS software. For each tooth, we measured the pre-operative socket height as the distance between the apex and lowest visible crestal point (Fig. **5**).

**Fig. (6):**
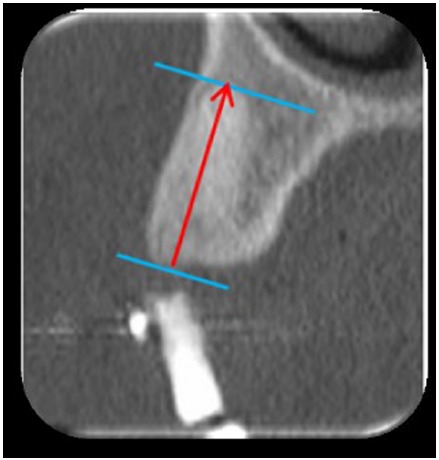
Three months after extraction, height is evaluated as the distance between apex and lowest crestal visible point on the postoperative CT Scan.

A computed tomography (CT) scan, Siemens^®^ Somatom Emotion (precision = 0.1 mm), was performed 3 months after the extractions for pre-implant planning. All of the CT scans were performed at Erasme hospital. For each socket, a measurement was performed between the apex and lowest visible crestal point (Fig. **6**).

Because of a panoramic radiography deformity and the fact that the pre- and post-extraction imaging techniques were different, a calibration was performed for each measure based on a three-rule using clear anatomical landmarks (adjacent tooth, or mandibular nerve). With the help of this calibration, the alveolar ridge preservation was measured between the apex of the socket and the lowest point of the alveolar crest of the socket at the crestal level.

### Statistical Analysis

The observed data were statistically analyzed using a Student’s t test for continuous (horizontal measurements) variable and Student’s t test for categorical (mono- or pluri-radicular teeth, smoking habits and immediate prosthesis) variables to assess bone loss 3 months after surgery. The measurements are presented as an average with a 95% standard deviation.

Statistical significance level was defined at P = 0.05.

A correlation test was applied for inter-observer measurements.

## RESULTS

The correlation coefficient between the two observers was r = 0.99425, with a 2-sided P-value of <.0001. This was considered to be a significant correlation. A mean average height loss of 0.72 mm (SD 0.71) (5.53% SD 5.19) was measured for all 95 extractions. No statistically significant difference in height loss (P = 0.982) was found between mono-radicular and pluri-radicular teeth (Table **1**).

Table **2** presents the bone loss measurements related to the type of teeth extracted. The mean difference in height before and after the extractions ranged from 0.6 to 0.8 mm. The highest alveolar height loss was measured for the upper canine (N = 12) (7.19% SD 6.80 bone loss), and the lowest height loss was observed for the lower incisors (N = 8) (4.17% SD 1.55 bone loss).

The average height loss after 3 months was 0.39 mm (SD 0.68) for patients who were smokers (N = 17) compared with 0.84 mm (SD 0.59) for non-smokers (N = 39). There was no statistically significant difference between smokers and non-smokers (P = 0.2). The average height loss after 3 months was 0.85 mm (SD 0.56) for patients who received an immediate prosthesis (N = 16) compared with 0.67 mm (SD 0.62) for those patients who did not receive an immediate prosthesis (N = 40) (P = 0.786), which was not statistically significant Table **3**.

## DISCUSSION

This study aimed at evaluating alveolar bone loss in the vertical dimension 3 months after tooth extraction when a mix of a particulate DFDBA 300 - 500 µm and platelet concentrates in the form of a gel was used as the filling material of the socket and an autologous fibrin membrane was used as a covering material.

For ethical reasons, no control group was used in this study, because alveolar ridge preservation was necessary in order to ensure adequate bone volume for implants [1-3].

Previous studies have shown that using height measurements to evaluate bone loss is possible by comparing pre- and post-operative standard radiographs [23, 24]. One of the restraints is that this does not authorize the evaluation of width loss. To measure height and width loss, pre- and post-extraction CT scans or cone beam CT should be performed, but there is an ethical restriction because of the radiation risks. The measurements of the bone height in our study were made on pre-extraction panoramic radiographs and 3-month post-extraction pre-implantation CT scans. To avoid any deformity in measurement, a three-rule calibration between the two types of radiographs was performed by the two independent operators for each measure, using anatomical landmarks.

In this study, focusing on 95 extractions, we observed a mean bone height loss of 0.72 mm (SD 0.71) after three months. For single rooted teeth (N = 72), this loss was, on average, 0.72 mm (SD 0.61), while for pluri-radicular teeth (N = 23), the loss was 0.73 mm (SD 0.74) (Table **1**).

In a systematic review, it was shown that the crestal height loss as assessed on the radiographs ranged from 0.8 to 3.6 mm, 3 to 6 months after tooth extraction if preservation techniques are not applied [5].

There is a great variability in the techniques, materials, and methods of measurement used in studies related to alveolar bone preservation, which renders it difficult to compare the results from different studies. A systematic review reported a 1.67 mm bone loss after a period of 3 to 12 months if an alveolar filling was applied, independent of the biomaterial used [1]. Another systematic review showed a statistically significant greater ridge reduction in bone height in a control group compared to the socket preservation group, with a weighted mean difference of 1.47 mm [4]. All of these studies concluded that the benefits of socket preservation therapies were demonstrated.

DFDBA in particulate form has been proposed as a suitable biomaterial in socket preservation [8-11]. In a histological study, it was demonstrated that DFBDA led to a statistically significant greater mean percentage of newly formed vital bone than FDBA [11].

The combination of DFDBA and platelet concentrates has been proven to be effective in the treatment of periodontal defects, but has never been evaluated in oral surgery in a large cohort group [25, 26]. In a RCT, the alveolar ridge height loss 4 months after extraction of intercalate anterior teeth was compared using pre- and post-extraction panoramic X-rays between 6 teeth that were DFDBA filled and 6 teeth that were DFDBA filled and covered by a palatal connective tissue [8]. In the DFDBA only group, a 0.72 mm height loss was observed compared with 0.86 mm in the DFDBA plus connective tissue group. These results are difficult to compare with our results because of the heterogeneity in study characteristics, differences in measurement techniques and socket covering, and the fact that the study had a small number of extractions. Our study included 95 extractions, and contrary to the previous study, we did not apply any exclusion criteria, except for the necessity to be over the age of 18 years. This suggests that the results gained with the technique of filling the extraction socket with a mix of particulate DFDBA 300 - 500 µm and platelet concentrates and covering it with an autologous fibrin membrane leads to an advantage in terms of the conservation of bone height. This is consistent with what the results of a clinical study, in which the mean height resorption 4 months after extraction and filling with platelet-rich-fibrin alone was 7.13% [18], which represents two percent greater loss than in our study. The more favourable results from our study could be because the platelet concentrates in the form of a gel and membrane could have acted *via* quicker healing of the overlaying gingival mucosa and hence may have secondarily preserved the filling mixture, which could explain the beneficial effects. Consistent with our results, in a recent systematic review, a meta-analysis was suggestive for a positive effect of platelet concentrates on bone formation in post-extraction sockets [19]. The beneficial effects of platelet concentrates are theoretically due to the high concentration of multiple growth factors that, as suggested in the literature, could remain active for more than 7 days in their fibrin matrix [12-14]. These growth factors are known to be essential for the regulation and stimulation of healing of soft and hard tissues by regulating cellular processes, such as mitogenesis, chemotaxis, and differentiation [12-14]. In a RCT of premolar extractions in 33 patients aimed at evaluating the impact of platelet-rich-fibrin on the quality of alveolar bone, it was shown that socket filling with a platelet concentrate led to better bone healing and quality in a statistically significant fashion [20]. In addition, it has been shown that platelet concentrates accelerated the healing of dermal soft tissue in a statistically significant fashion and that they had a strong stimulant effect on capillary regeneration in oral wound healing during the early stages of wound healing [16, 27].

In our study, the average height loss after 3 months was 0.39 mm (SD 0.68) for patients who were smokers (N = 17) compared with 0.84 mm (SD 0.59) for non-smokers (N = 39), which was not statistically significant (P = 0.2). Although the relationship between tobacco use and post-extraction alveolar height loss has not been proven, it has been suggested that smoking habits may adversely affect wound healing and lead to a higher degree of complications, such as alveolitis or infections [28]. In our study, the socket filling material was protected by a fibrin membrane at the time of the early healing period, which could have contributed to the good results, as well as the degree of accelerated soft tissue healing due to the platelet concentrates. We did not find any significant difference in height loss between patients who received an immediate prosthesis (N = 16) or who did not receive an immediate prosthesis (N = 40) (P = 0.786). Those who received prostheses lost 0.85 mm (SD 0.56) on average, and those who did not receive prostheses lost 0.67 mm (SD 0.62). There is no evidence in the literature as to the effect of immediate provisional prostheses on extraction socket healing. We can assume that when a removable prosthesis is delivered immediately after extractions, it may have a role in soft tissue protection, which may contribute to a good result in terms of height bone preservation, but on the contrary, it may constitute a factor of mechanical restriction for soft and hard tissue healing. Further studies would be needed to answer this question.

## CONCLUSION

This clinical radiological retrospective study aimed at evaluating the height of bone loss in the vertical dimension 3 months after extractions after use of a technique for alveolar preservation using a mix of a particulate demineralized freeze-dried bone allograft 300 - 500 µm and platelet-rich-fibrin in the form of gel to fill the socket and an autologous platelet-rich-fibrin membrane to cover it.

The mean average height loss is 0.72 mm (SD 0.71), which represents 5.53% (SD 5.19) of the initial height, with no significant influence for tobacco or provisional prosthesis.

There is a good potential for the use of particulate demineralized freeze-dried bone allograft 300 - 500 µm associated with platelet concentrates in the form of a gel and membranes in alveolar bone preservation.

Further clinical prospective studies should be conducted to better evaluate the potential benefits of this technique.

## Figures and Tables

**Table 1:** Mean bone loss in height 3 months after dental extraction.

	**N**	**Mean (SD) height loss** **3 months** **after extraction (mm)**	**Mean height loss** **3 months** **after extraction (%)**	**P-value** ^a^
Mono-radicular teeth	72	0.72 (0.61)	5.45 (4.41)	0.982
Pluri-radicular teeth	23	0.73 (0.74)	5.64 (5.42)	
Total	95	0.72 (0.71)	5.53 (5.19)	

^a^ Statistical significance level was defined at P = 0.05. N = total number of tooth extracted, with mono-radicular and pluri-radicular teeth. t-test was performed to compare the mean bone loss between mono- and pluri-radicular teeth.

**Table 2:** Mean bone loss in height related to the type of tooth.

	**Number of** **teeth extracted**	**Mean (SD) height loss** **3 months** **after extraction (mm)**	**Mean (SD)height loss** **3 months** **after extraction (%)**
Upper incisor	22	0.6 (0.61)	6.3 (5.06)
Upper canine	12	0.8 (0.73)	7.19 (6.80)
Upper premolar	31	0.8 (1.11)	5.91 (6.71)
Upper molar	11	0.7 (0.74)	5.38 (5.42)
Lower incisor	8	0.5 (0.17)	4.17 (1.55)
Lower canine	2	0.6 (0.07)	4.57 (0.61)
Lower premolar	7	0.5 (0.51)	4.28 (6.47)
Lower molar	2	0.6 (0.84)	5.9 (6.07)

SD = standard deviation. Mean bone loss for each type of teeth. The measurements are presented as an average with a 95% standard deviation.

**Table 3:** Bone loss in height 3 months after dental extraction according to smoking habit or provisional prosthesis.

	**Number of** **teeth extracted**	**Mean (SD) height loss** **3 months** **after extraction (mm)**	**Mean height loss** **3 months** **after extraction (%)**	**P-value** ^a^
Smokers	17	0.39 (0.68)	4.06 (6.51)	0.2
Non smokers	78	0.84 (0.59)	6.23 (5.49)	
Immediate prosthesis	16	0.85 (0.56)	5.18 (3.17)	0.786
No immediate prosthesis	79	0.67 (0.62)	6.53 (6.47)	

^a^ Statistical significance level was defined at P = 0.05. Number of teeth extracted for each categories of patient, and the mean bone loss according to the smoking habit or provisional prosthesis. Student’s t test was performed to determine if tobacco use or the provisional removable prosthesis had any effect on bone loss.
